# Social egg freezing: a reproductive chance or smoke and mirrors?

**DOI:** 10.3325/cmj.2015.56.387

**Published:** 2015-08

**Authors:** Lucia Martinelli, Lucia Busatta, Lucia Galvagni, Cinzia Piciocchi

**Affiliations:** 1 MUSE – Science Museum, Trento, Italy *lucia.martinelli@muse.it*; 2 BioLaw Project, Faculty of Law, University of Trento, Trento, Italy; 3 Center for Religious Studies, Fondazione Bruno Kessler, Trento, Italy

Autologous human oocyte cryopreservation allows storing woman’s eggs to be used later by the same donor when therapeutic or elective reasons induce women to postpone motherhood. In the case of non-medical motivation, this practice is termed social freezing. By shifting the awareness from a medical procedure to social relations, frozen oocytes are becoming polemic bio-objects, which pose new questions about timing related to fertility decline and motherhood, and point out the ambiguous interpretation of biology innovations as promoter of new opportunities or new facade of enduring contradictions.

## Assisted reproductive technologies: a looking glass to analyze society

Within the last 30 years, assisted reproductive technologies (ARTs) have been successfully developed to overcome infertility and have become outstanding examples of biomedical innovations influencing our lives with their heavy load of promises and controversies. As these technologies grow more sophisticated, they raise new questions concerning the possibility to medically manipulate lives and bodies (in particular of women) and to intrude in couple’s intimacy and social relations. This controversial interaction between technology and society, previously described as bio-objectification (1), shifts the attention from a medical procedure to a social phenomenon, which needs to be analyzed within the framework of bio-ethics, bio-policy, bio-economy, and bio-law (2). In this complex interaction, technical aspects intermingle with cultural issues and the anthropological, psychological, and social meaning of parenthood and kinship. In particular, the split between sexuality and reproduction and between biological and social parenthood, as well as the intervention of new and different actors in the matter (medical professionals, gamete donors, surrogate mothers, etc) enlighten the meaning of reproductive roles and demand the elaboration of new definitions for gender, sex, person, family, parenthood, and offspring. This new scenario requires us to re-consider and re-define (also from the legal point of view) the connections between the subjects involved and their rights.

The legal regulation of ARTs varies among countries. The European Convention of Human Rights protects some very basic rights, and the case-law of the Strasbourg Court has affected some national regulations (ie, S.H. and other v. Austria, appl. No. 57813/00, First Section, 1 April 2010 and Grand Chamber, 3 November 2011; Costa and Pavan v. Italy, appl. No. 54270/10, 28 August 2012). Similarly, each State in the US has its own regulation on ARTs, but we could argue that the main trend there is far more oriented for a *laissez faire* regime, rather than for a strict procedural or substantial regulation (*Assisted Reproduction*, at http://www.thehastingscenter.org/Publications/BriefingBook/Detail.aspx?id=2210; G12 Country Regulations of Assisted Reproductive Technologies, 2010, at *https://cbhd.org/content/g12-country-regulations-assisted-reproductive-technologies*).

Due to these features, ARTs seem to be suitable tools “to observe and interpret our own reality, to stand back and reframe traditional questions or to pose new ones” (3).

## From medical oocyte cryopreservation to social egg freezing

The phenomenon of oocyte cryopreservation (egg freezing) is an interesting tool to analyze critical issues in contemporary societies. This techno-medical innovation, which appeared in the late 1980s, is nowadays efficiently used to store and preserve viable oocytes after collection when ovaries are still sufficiently “young” to enable proper gamete production and collection (4-6), as human oocyte banks have been established worldwide and hundreds of babies have been already born (4). Alike the human embryo, stored oocytes can be characterized as bio-objects, ie, they call into question and destabilize traditional definitions and boundaries such as natural/artificial and life/non-life, may be separated from a body and kept “alive” to be used later, manipulated, stored, and mobilized (exchanged, donated, sold), they produce hope and controversy, and require specific policy, regulation, and communication principles (7-9).

Autologous human oocyte cryopreservation allows storing women’s eggs for their later use by the same woman from whom they were retrieved, while heterologous fertilization implies the donation of eggs to another woman for her pregnancy. In this article, we will focus on the first concept. Autologous human oocyte cryopreservation has been used in association with in vitro fertilization by individuals who object to freezing their embryos because of ethical/religious beliefs (10) or wish to bypass legal restrictions against egg donation (11). Moreover, this strategy has been recognized as an opportunity to postpone motherhood, first of all when medical constraints discourage pregnancy, such as before starting medical treatments with deleterious effects on reproduction (for instance cancer treatments, as chemo- and radio-therapy) (12), in the case of fertility preservation in women with family history of early menopause (13), or in the case of a sex change operation (14). This opportunity could also be given to women at risk of injury to reproductive organs, or death, as in the case of members of Armed Forces in war zones (Fertility Center of California, *http://www.spermbankcalifornia.com/military-sperm-bank.html*). Finally, egg freezing can be adopted when women wish to postpone motherhood due to various personal problems, such as educational or career demands, or absence of a partner or a stable relationship.

Oocyte cryopreservation has also recently been defined as a sort of “reproductive insurance” against age-related infertility (15,16), but on the other hand it promotes a medicalization of a state in which future infertility is predicted. For instance, the Israel National Bioethics Council considers age-related fertility decline to be a medical problem, and recommends treating egg freezing as a preventive medicine strategy (6).

Reasons to undergo egg cryopreservation to prevent fertility decline, due to either illness or aging, have sometimes been differently depicted: as the only option (thus deserving help) or as a life-style choice (thus deserving skepticism or even dissent) (11,17). The latter has been termed as social freezing (18,19). In the case of non-medical reasons, women have been frequently described as perpetrators or victims, respectively, being either selfish career women or preys of a profit-oriented (male) medicine, which takes advantage of women’s infertility fear (11,5), or even victims of a male-oriented workplace, in which social and welfare benefits for maternity leaves, when provided, are not properly effective, as also pointed out by the European Parliament (20).

## Balancing job and family choices

It might be expected that social freezing would be viewed as a business opportunity. Some companies are even offering financial plans to support women to freeze their oocytes in a venture which could turn out to be quite expensive (eg, in Europe and USA respectively: Ovita, Bregenz, Austria, *http://www.ovita.eu/en-us/eizelleneinfrieren.aspx* and EggBanxx, USA, *https://www.eggbanxx.com/financing-egg-freezing*). Yet, we are facing the launch of a new wave, which began in 2014, when the two Silicon Valley giants Facebook and Apple announced that they would cover the expenses for egg freezing for non-medical reasons for their employees, as an additional benefit aiming at conciliating motherhood with high-powered careers (21). Accordingly, social freezing paid by the employer has been launched by these companies as an opportunity for “glass-ceiling breakers” (22). The spread of this news, nonetheless, is raising a controversial debate around the world. It should be questioned if this policy, instead of a positive action, is a new constrictive measure preventing the adoption of more flexible structural changes in the work organization to facilitate conciliation between career and motherhood. In addition, it could be interpreted as an intrusive measure into female employees’ procreative choices.

This circumstance points out that there is still a lack of adequate structural organization in the workplaces to incentive career and support motherhood. To manage the incongruity between proper physiological age and postponed motherhood, social freezing has increasingly been proposed as a new opportunity to conciliate personal and professional needs and pregnancy. It should rather be questioned if such needs should be better regarded as societal problems, which would deserve proper changes in social organization. If we cannot force anyone to have babies at a specific age, we should enhance awareness of the most suitable reproductive conditions and timing. Concurrently, we should also modify social and work conditions to allow women and men to make life choices and decisions where personal and professional projects can be harmonized. This should necessarily imply the development of structures and work dynamics more oriented to the parenthood needs.

## Racing against time

Among the various aspects connected to the complex issue of social freezing, the time factor emerges as a relevant variable. Cryopreservation allows a part of a body to rest in a quiescent state waiting for “the right moment” to undertake its function and the suggested standard period is 10 years (Human Fertilisation and Embryology Authority, London, UK, *http://www.hfea.gov.uk*). To be viable, moreover, oocytes need to be collected from “young” ovaries to prevent their quality and quantity decline, which increases with aging. Success rates of in vitro fertilization performed with young heterologous oocytes in older women (44 and above), for instance, suggest that egg age and quality is more relevant for successful in vitro fertilization than the age of the patient (23). It has been also reported that the rates of survival, fertilization, and implantation of “young” cryopreserved oocytes fertilized with intracytoplasmic sperm injection are comparable with those of fresh oocytes (17). For these reasons, scientific literature recommends to “freeze in time” oocytes to properly preserve woman’s fertility potential (23).

The concept of timing related to successful freezing is particularly stressed in the communication targeted to engage potential users, such as on the websites of clinics that provide this service. According to internet search, a recurring expression in the narrative to motivate egg freezing refers to a future when potential clients will be ready for something. Thus, timing can involve a *status quo*, as in the case of “women who may not be in a secure relationship” (The London women’s clinic, London, UK, *http://www.londonwomensclinic.com/london/egg_freezing*) or waiting for “Mr. Right” (Eggsurance Inc.^TM^, USA, *http://www.eggsurance.com/)*. Interestingly, this latter expression may evoke the Prince Charming from the fairy tales and puts women in the role of inescapable subordination and passivity (24). Concerning aging, this technique is constantly proposed as a tool to “stop [the] biological clock” with the final aim of reconciling “personal and professional timelines” (Pacific Fertility Center, San Francisco, CA, USA, *http://www.pacificfertilitycenter.com/* or Extend fertility, USA, *http://www.extendfertility.com/*), because “since Einstein and social egg freezing we have learned that time is relative” (Ovita, Bregenz, Austria, *http://www.ovita.eu/en-us/eizelleneinfrieren.aspx*) and because “when it comes to issues of fertility, it is the age of the egg, not the age of the woman that matters most” (Extend fertility, USA *http://www.extendfertility.com/*). For egg cells, however, timing is a relevant constraint too, since “aging of human eggs cannot be halted or reversed” and “egg quality decline begins at age 30 and increases significantly after age 35” (Eggsurance Inc.^TM^, USA), thus social freezing is constantly recommended to be planned well in advance.

Thus, the main question could be: is there an adequate time to have children? When is it? And who should decide it? For answering these challenging questions, the cultural component in the overall issue has to be considered, which also includes ethical aspects and worldwide regulations on ARTs. It is important to remember that as human beings we experiment and live limits as our main condition: this happens in biological terms, in terms of reproductive capabilities, and the available time in which we have to realize our reproductive projects and plans. From a socio-economical viewpoint, the choice to postpone pregnancies might also have an impact upon the society as a whole: motherhood at an older age could be more demanding, for the woman, the unborn, and for the health care organization. These factors, characterized by high level welfare benefits, should be adequately taken into considerations, as the costs of risky pregnancies might become a burden for the society as a whole. This is also true when the costs for egg freezing could be paid individually as the final aim of this intervention is to try to have a pregnancy at a stage of life when serious problems of fertility and risky pregnancy may arise. Should limits be set by law-makers? Should the matter be left to professional regulation and guidelines? The choice might depend upon the legal order concerned, it could be either political, ethical, or medical in nature; nevertheless, it should be remarked that, in any case, both law-makers and medical doctors must comply with those limits imposed by “nature” and by the current development of sciences and technologies, jointly considered.

## The information gap

In Western countries, especially in groups with higher education, the trend to postpone motherhood until the fourth decade of life for a series of elective reasons is becoming increasingly common (25), resulting in likely unwanted infertility. The risk of permanent biological childlessness decreased from 6% to 35% when women delayed pregnancy until the age of 30 and 40, respectively (17). This risk seems to be underestimated or ignored by younger generations (26-29), who seem to have an “ambivalent” attitude toward parenthood, which has been interestingly termed as “perpetual postponing” (17). In this framework, ARTs and, most recently, social freezing are perceived as suitable solutions. This even leads to founding of ad hoc parties (Egg freezing party, San Ramon, CA, USA, *http://eggfreezingparty.com/*), which engage younger women in oocyte preservation. Despite such reports in favor of the efficacy and safety of oocyte cryopreservation, it should be considered that the success rate frozen eggs in ARTs varies from clinic to clinic, and different statistics, and thus different optimistic visions, are reported in literature and on the websites of the various fertility clinics. According to Human Fertilisation and Embryology Authority, London, UK (*http://www.hfea.gov.uk*), in United Kingdom up to December 2012 around 18 000 eggs have been stored. Among the 580 embryos created from these for autologous treatments, there were only 20 live births, thus resulting in a 12.5% chance that woman will have a baby after a cycle of frozen egg in vitro fertilization. On the other hand, an assisted reproduction treatment at 42 years of age would result in a live-birth rate of 6.6% per cycle with own fresh eggs (17), while the equivalent rate with own oocytes cryopreserved at age 30 would be >40% per transfer. It should be pointed out, thus, that public awareness and knowledge about social freezing seem to be quite inadequate; complete and scientifically sound information on outcomes, success, and unsuccessful rates and risks connected to postponed pregnancies and about the limits of assisted reproduction should be duly made available to women, as well as to young generations.

Worth stressing, information is the key element for a proper informed consent, an issue that may also cause further legal problems (16). Even those in favor of social egg freezing state that correct and complete information concerning success rates and maternal risks should be provided to women (15). Therefore, we could argue that without all necessary information, the woman’s choice to undergo egg freezing is not fully free and responsible: on the contrary, women could be even considered unaware victims of “a commercially exploitative context, thus undermining rather than expanding reproductive autonomy” (16).

## Conclusion

Our essay points out once more that the so-called social egg freezing is a paradigmatic demonstration of how the medicalization of women’s bodies can be used to mask social and cultural anxieties about aging, illness, reproduction, singlehood, and risk (11). Frozen oocytes become polemic bio-objects that raise questions about the interpretation of the impact and meaning of such medical technology: can this improve awareness about reproductive choices and health, thus resulting in a useful tool for enhancing (women’s) life quality, or is this just a new practice for conserving enduring contradictions?

It should also be remarked that, within contemporary societies, the choice to procreate involves public policy issues that range from labor market regulations to social benefits for families with children. Therefore, instead of encouraging the view that social freezing to postpone parenthood would be a suitable alternative to conventional reproduction, a more inclusive society should find concrete structural solutions for supporting young families and helping women to conciliate motherhood with professional activity.

Finally, in our societies, female bodies tend to be transformed into “public spaces,” where life and fetuses become abstract objects, unrelated to women (30). This seems also to be true for frozen eggs if the use of this technology, instead of an informed choice, becomes a “fashionable” trend, well publicized by the social media, or the outcome of an employer’s controversial offer. Women, on the other hand, still remain the necessary –and main actors– to decide about the pregnancy and giving birth. This also relates to the vision of what should be the right time to have children, which should undeniably be the result of a free and conscious choice.
